# The Role of GLP1 in Rat Steatotic and Non-Steatotic Liver Transplantation from Cardiocirculatory Death Donors

**DOI:** 10.3390/cells8121599

**Published:** 2019-12-09

**Authors:** Cindy G. Avalos-de León, Mónica B. Jiménez-Castro, María Eugenia Cornide-Petronio, Araní Casillas-Ramírez, Carmen Peralta

**Affiliations:** 1 Institut d’Investigacions Biomèdiques August Pi I Sunyer (IDIBAPS), 08036 Barcelona, Spain; avalosdl.cin@gmail.com (C.G.A.-d.L.); monicabjimenez@hotmail.com (M.B.J.-C.); cornide@clinic.cat (M.E.C.-P.); 2 Hospital Regional de Alta Especialidad de Ciudad Victoria “Bicentenario 2010”, Ciudad Victoria 87087, Mexico; 3 Facultad de Medicina e Ingeniería en Sistemas Computacionales de Matamoros, Universidad Autónoma de Tamaulipas, Matamoros 87300, Mexico; 4 Centro de Investigación Biomédica en Red de Enfermedades Hepáticas y Digestivas (CIBERehd), 08036 Barcelona, Spain

**Keywords:** cardiocirculatory death, GLP1, liver transplantation, steatotic liver grafts, ischemia-reperfusion damage

## Abstract

In liver transplantation (LT), organ shortage has led to the use of steatotic and non-steatotic grafts from donors after cardiocirculatory death (DCD). However, these grafts, especially those with steatosis, exhibit poor post-operative outcomes. To address this problem, we investigated the roles of gut-derived glucagon-like peptide 1 (GLP1) and dipeptidyl peptidase 4 (DPP4), the serine protease that cleaves it, in steatotic and non-steatotic LT from DCDs. Using Zucker rats, liver grafts from DCDs were cold stored and transplanted to recipients. GLP1 was administered to donors. The levels of GLP1 in intestine and of both GLP1 and DDP4 in circulation were unaltered following cardiocirculatory death (CD). In steatotic livers from DCD, increased GLP1 and decreased DPP4 were recorded, and administration of GLP1 caused a rise in hepatic GLP1 and a reduction in DDP4. This protected against inflammation, damage, and proliferation failure. Conversely, low GLP1 and high DDP4 were observed in non-steatotic livers from DCD. The exogenous GLP1 did not modify hepatic DDP4, and the accumulated GLP1 exerted harmful effects, increasing damage, inflammation, and regeneration failure. Herein, we show that there are differences in GLP1/DDP4 regulation depending on the type of liver implanted, suggesting that GLP1 can be used as a novel and effective therapy in steatotic grafts from DCDs but that it is not appropriate for non-steatotic DCDs.

## 1. Introduction

The critical shortage for liver transplantation (LT) has been addressed by means of extending the criteria for suitability. The resulting grafts, which include steatotic livers and livers from donors after cardiocirculatory death (DCD), exhibit accentuated ischemia-reperfusion (I/R) injury, which increases the risk of primary graft non-function and early graft dysfunction [1].

Recent investigations in animal models and humans have focused on the relevance of the gut–liver axis to solve pathologies associated with the inflammatory processes involved in hepatic or ileal diseases [2]. Glucagon-like peptide 1 (GLP1) is a peptide synthesized by the small intestine. GLP1 is then secreted and released into the circulation via portal vein, thus reaching the liver [3]. It has been reported that GLP1 exhibited protective effects against ischemia-reperfusion (I/R) damage, since treatment with a GLP1 analog resulted in anti-inflammatory actions and inhibition of cell death in non-steatotic rats undergoing hepatic partial warm ischemia [4]. To the best of our knowledge, there are no studies of GLP1 effects in experimental models of LT using DCD organs, either non-steatotic or steatotic. Gut-derived GLP1 is notoriously rapidly degraded by dipeptidyl peptidase 4 (DPP4) in the circulation, limiting its half-life [5]. Under physiological conditions, only a fraction of intestinal GLP1 reaches the liver in its active form due to the capillaries expressing the enzyme DPP4, which degrades GLP1 rapidly [6]. Also, the liver is an organ with high DPP4 expression, which is influenced by hypoxia, and DPP4 levels are increased in inflammatory liver diseases [7].

In the present research, we focus on the gut–liver axis to investigate whether damage, inflammation, and proliferative failure in an experimental model of transplantation in rats with liver grafts from DCDs are affected by changes in the hepatic levels of gut-derived-GLP1 and/or up-regulation of hepatic DPP4 expression. We try to pharmacologically increase hepatic GLP1 levels through administering intravenously via porta vein the recombinant native and active form of GLP1 (7–36) in donor rats. We also investigate whether the possible effects of GLP1 in LT with non-steatotic and steatotic livers from DCDs can be exerted through regulation of inflammatory response. Thus, suppressors of cytokine signaling (SOCS1, 2, and 3) are assessed, given that they are key mediators in regulating inflammatory response and necrosis. Indeed, it has previously been suggested that the anti-inflammatory effects of GLP1 are exerted through regulation of SOCS1, 2, and 3 [8].

All these investigations could promote not only the generation of new scientific knowledge about the as yet unidentified pathophysiological mechanisms in steatotic and non-steatotic LT from DCDs but could also help to identify effective strategies for addressing this pathology in a scenario in which both cardiocirculatroy death (CD) and steatosis are major risk factors in LT.

## 2. Materials and Methods

### 2.1. Experimental Animals

This study was performed using homozygous [obese, (Ob, *fa/fa*), strain code 185] and heterozygous [lean (Ln, *fa/+*), strain code 186] male Zucker rats (Iffa Credo, France) aged between 10 and 11 weeks. Ob rats show moderate macrovesicular and microvesicular fatty infiltration in hepatocytes (40% to 60% steatosis), whereas Ln rats show no evidence of steatosis [9]. All procedures were approved by the Laboratory Animal Care and Use Committee of Barcelona University and by the government of Catalonia (Generalitat de Catalunya, DAAM 9353). European Union regulations (Directive 86/609 EEC) for animal experiments were respected.

### 2.2. Cardiocirculatory Death Induction

Animals were anesthetized by isoflurane, and heparin sodium solution was administered to donors. A well-established rodent CD model was selected: induction of CD by hypoxia through incision of the diaphragm and blocking the descending aorta [10,11]. The in situ warm ischemia period was 45 min.

### 2.3. Surgical Procedure of Liver Transplantation

Steatotic and non-steatotic liver grafts from DCDs or non-DCDs were flushed and stored in University of Wisconsin (UW) solution for 6 h. Standard orthotopic LT in Ln Zucker rats was performed according to the double cuff technique [9,12]. The anhepatic phase duration was between 17 and 20 min.

### 2.4. Experimental Design

Animals were randomly distributed to form the following groups:

**Group 1. Sham** (n = 12). Six Ob and six Ln Zucker rats were anesthetized and maintained for 45 min.
**Group 2. CD** (n = 12). Six Ob and six Ln Zucker rats were anesthetized. After cardiocirculatory death (CD) induction, rats were maintained in situ in warm ischemia for 45 min [10,11].
**Group 3. LT** (n = 24, 12 transplantations). Six Ob and six Ln Zucker rats were anesthetized and maintained for 45 min. Then, steatotic and non-steatotic liver grafts were flushed, isolated, and stored in ice-cold UW solution for 6 h. Finally, they were implanted in 12 Ln Zucker rats [9].
**Group 4. CD+LT** (n = 24, 12 transplantations). Six Ob and six Ln Zucker rats were anesthetized. After the induction of CD, the animals were maintained in situ in warm ischemia for 45 min. Then, steatotic and non-steatotic liver grafts were flushed, isolated, and stored in ice-cold UW solution for 6 h. Finally, they were implanted in 12 Ln Zucker rats [9,10,11].
**Group 5. CD+GLP1+LT** (n = 24, 12 transplantations). As group 4, but donors were treated with GLP1 [glucagon-like peptide 1 (7–36) amide] infusion into the portal vein at a rate of 1 pMol × kg × min, 10 min before CD. Then, steatotic and non-steatotic liver grafts were flushed, isolated, and stored in ice-cold UW solution for 6 h. Finally, they were implanted in 12 Ln Zucker rats [13].

Plasma, liver, and intestine samples were collected 4 h after LT (groups 3, 4, and 5) before the induction of CD and before the harvesting of liver grafts from donors (groups 1 and 2). For the current study, conditions (including duration of cold ischemia and reperfusion) [9,10,11], drug dosage, and timing of GLP1 infusion used in the present study were selected based on a previous study [13] as well as on preliminary studies by our group. Before the selection of dose and pre-treatment times of GLP1 used in the current study (1 pMol × kg × min, 10 min before CD, group 5), we performed experimental groups based on administration of GLP1 infusion at the doses of 0.2, 1, 5, 25, and 100 pMol × kg × min in donor rats at different pre-treatment times (10, 30, or 60 min) before induction of CD, and the effects on hepatic injury and regeneration were determined 4 h after LT in recipient rats.

### 2.5. Biochemical Assays

Plasma transaminases [alanine aminotransferase (ALT) and aspartate aminotransferase (AST)] were measured photometrically using standard procedures. Von Willebrand Factor (vWF) (as an index of cell endothelial damage) and total bilirubin were measured in plasma by an enzyme-linked immunosorbent assay (Elabscience Biotechnology, Houston, TX, USA and MyBiosource, San Diego, CA, USA, respectively) according to the manufacturer’s instructions. Nitrotyrosine was used as an index of peroxynitrite (ONOO^−^) formation, a potent oxidant generated from the reaction of the superoxide (O_2_^−^), with the nitric oxide (NO). Nitrosylated proteins were measured using a commercial kit (Hycult Biotechnology BV, Uden, Netherlands), and NO production was determined by the accumulation of nitrites and nitrates using a spectrophotometric method according to the manufacturer’s instructions (Cayman Chemicals, Ann Arbor, MI, USA) [9,14].

Lipid peroxidation was determined by measuring the formation of malondialdehyde (MDA) as an indirect index of the oxidative injury induced by the ROS [15]. Briefly, 0.5 mL of 0.5% butylated hydroxytoluene was added to 2 mL of intestine or liver homogenate to prevent lipid autoxidation. For protein precipitation, 2 mL of 20% trichloroacetic acid was added to 2 mL of homogenate. After mixing and centrifuging, 1 mL of 0.67% thiobarbiturate solution was added to the supernatant and boiled for 60 min. After cooling, optical density at 530 nm was assayed [15].

Myeloperoxidase (MPO), as an index of neutrophil accumulation, was measured photometrically using tetramethyl-benzidine as a substrate. Intestine and liver samples were macerated with 0.5% hexadecyltrimethylammonium bromide in 50 mM phosphate buffer pH 6.0. Homogenates were then disrupted for 30 s using a sonicator at 20% power and subsequently snap frozen in dry ice and thawed on three consecutive occasions before a final 30 s sonication. Samples were incubated at 60 °C for 2 h and then spun down at 4000 *g* for 12 min. Supernatants were collected for the MPO assay. Enzyme activity was assessed photometrically at 630 nm. The assay mixture consisted of 20 μL supernatant, 10 μL tetramethylbenzidine (final concentration 1.6 mM) dissolved in dimethyl sulfoxide, and 70 μL H_2_O_2_ (final concentration, 3.0 mM) diluted in 80 mM phosphate buffer pH 5.4. An enzyme unit is defined as the amount of enzyme that produces an increase of one absorbance unit per minute [16].

### 2.6. Western Blotting

Liver and intestinal tissues were homogenized in an ice-cold radioimmunoprecipitation assay (RIPA) buffer with protease and phosphatase inhibitors (Sigma Aldrich, St Louis, MO, USA). Samples were sonicated for 15 s at 60 w and centrifuged at 20,000× *g* for 10 min. Plasma, liver, and intestine homogenates containing an equal amount of protein were mixed in Laemmli loading buffer and separated on a sodium dodecyl sulfate (SDS-PAGE) 8–12% poly-acrylamide gel electrophoresis and transferred to polyvinylidene fluoride (PVDF) membranes. After assessing transfer, the membranes were saturated in 4 mM Tris-HCl, pH 7.6, 30 mM NaCl, and 0.1% Tween 20 (TBST) containing 5% non-fat milk and incubated overnight at 4 °C using antibodies against GLP1 (ab23468), cyclin A (sc-239), CD-26 (also known as DPP4; sc-52642), SOCS3 (sc-9023) and transferrin (sc-52256) (Santa Cruz Biotechnology, Dallas, TX, USA), SOCS1 (ARP42148) and SOCS2 (ARP63434) (Aviva Systems Biology, San Diego, CA, USA), cyclin D1 (SAB5500090), and β-Actin (A2228) (Sigma-Aldrich). Immunoreactive protein bands were visualized using an enhanced chemiluminescence kit using peroxidase-conjugated secondary antibodies and Clarity™ Western ECL Substrate (Bio-Rad Laboratories, Hercules, CA, USA). Blots were subsequently “stripped” and incubated with antibody against β-Actin for tissue and transferrin for plasma, respectively, as an internal control for protein loading. The scanning values for liver GLP1, DPP4, cyclin D1, cyclin A, SOCS1, SOCS2, and SOCS3 were divided by the scanning values for β-Actin and the scanning values for plasma GLP1 and DPP4 by the scanning values for transferrin. The values were quantified with scanning densitometry software (Quantity One; Bio-Rad Laboratories).

### 2.7. Liver and Intestine Histology

To assess the severity of hepatic injury, paraffin-embedded liver sections were stained with hematoxylin and eosin, and blind histological scoring was performed by a board certified pathologist using a point-counting method on an ordinal scale, as follows: grade 0, minimal or no evidence of injury; grade 1, mild injury consisting of cytoplasmic vacuolation and focal nuclear pyknosis; grade 2, moderate to severe injury with extensive nuclear pyknosis, cytoplasmic hypereosinophilia, and loss of intercellular borders; grade 3, severe necrosis with disintegration of hepatic cords, hemorrhage, and neutrophil infiltration; grade 4, very severe necrosis with disintegration of hepatic cords, hemorrhaging, and neutrophil infiltration [9]. The severity of intestinal damage was scored on a scale from 0 to 5, as described by Chiu et al. [17]. Liver steatosis was evaluated with red oil staining of frozen specimens, and the percentage of steatosis was calculated by image analysis according to the standard procedure.

### 2.8. Immunohistochemistry

After fixation with 4% formalin/phosphate-buffered saline, paraffin-embedded livers were sliced and immunostained using mouse monoclonal antibody anti-proliferating cell nuclear antigen (PCNA) (M0879) (Agilent, Santa Clara, CA, USA). Staining was developed with diaminobenzidine (DAB), using DAKO Envision System (Agilent) according to the manufacturer’s protocol. Sections were counterstained with hematoxylin. At least 30 high-power fields were counted per slide.

### 2.9. Statistics

Data are expressed as means ± standard error and were statistically analyzed via one-way analysis of variance, followed by post hoc Student–Newman–Keuls test. *p* < 0.05 was considered significant.

## 3. Results

### 3.1. GLP1 and DPP4 in Recipients of Steatotic and Non-Steatotic Liver Grafts from DCDs

**Figure 1:**
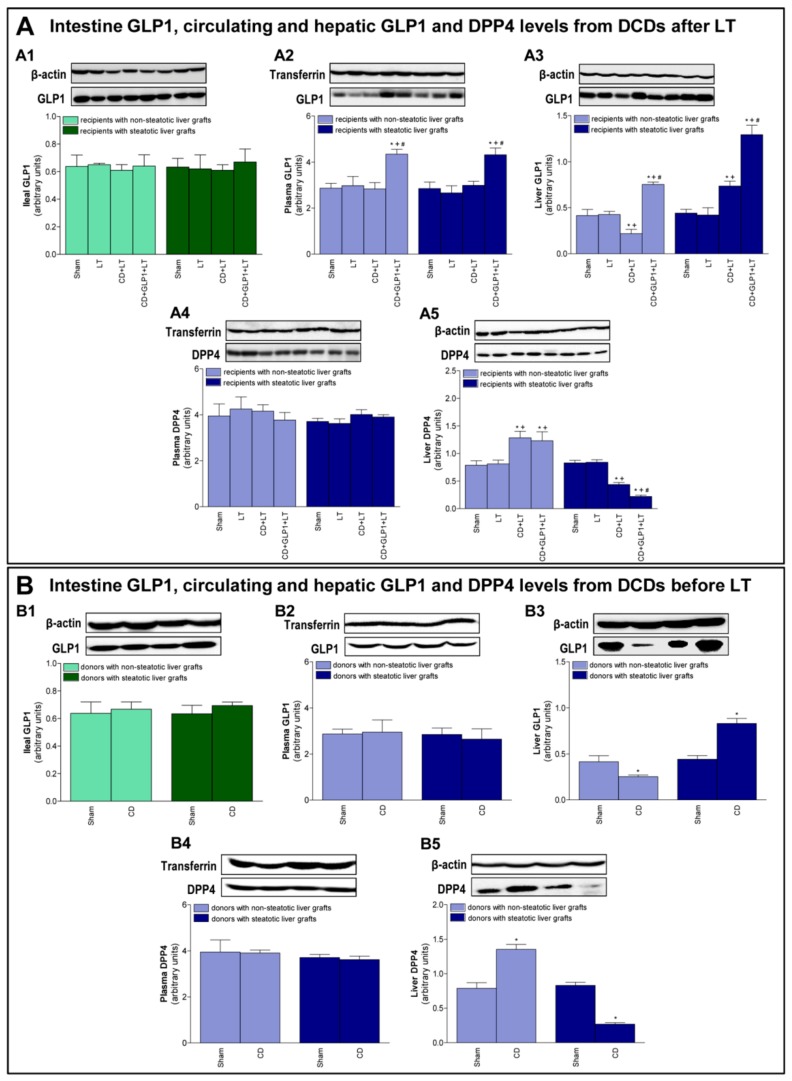
Glucagon-like peptide 1 (GLP1) and dipeptidyl peptidase 4 (DPP4) levels. (**A**) GLP1 protein expression in intestine (**A1**), plasma (**A2**) and liver (**A3**) and DPP4 protein expression in plasma (**A4**) and liver (**A5**) in recipients of non-steatotic and steatotic liver grafts from cardiocirculatory deaths (DCDs) (for A1–A4, six transplants per group were performed for each measurement). (**B**) GLP1 protein expression in intestine (**B1**), plasma (**B2**), and liver (**B3**), and DPP4 protein expression in plasma (**B4**) and liver (**B5**) in non-steatotic and steatotic donors before procurement of liver grafts from DCDs [for B1–B4, six lean (Ln) and six obese (Ob) animals per group in each measurement]. Representative western blots for the proteins GLP1 and DPP4 and the housekeeping protein β-actin for tissue and transferrin for plasma are shown at the top, and densitometric quantification of GLP1 and DPP4 protein expression normalized to β-actin for tissue and transferrin for plasma, respectively (arbitrary units) is shown at the bottom. * *p* < 0.05 vs. Sham; + *p* < 0.05 vs. liver transplantation (LT); # *p* < 0.05 vs. CD+LT.

In both CD+LT and LT groups, intestinal and plasmatic levels of GLP1 were similar in recipients of either non-steatotic or steatotic grafts. However, in the CD+LT group, decreased levels were detected in non-steatotic grafts and increased levels in steatotic grafts when compared to the results of the LT group. We then evaluated protein expression of DPP4, as it is well established that DPP4 is abundantly expressed in the liver and has a role in the degradation of GLP1 [6]. In both CD+LT and LT groups, plasmatic levels of DPP4 were similar in recipients of either non-steatotic or steatotic grafts. Results indicated, however, that in the CD+LT group, increased levels were recorded in non-steatotic grafts and decreased levels in steatotic grafts when compared to the LT group. Thus, in liver, DPP4 levels were inversely correlated with GLP1 levels (Figure 1A).

### 3.2. GLP1 and DPP4 Levels in Donors Before Procurement of Liver Grafts from DCDs

The same changes in GLP1 and DPP4 recorded in recipients after transplantation (Figure 1A) were also observed in donors immediately after the CD induction and just before the procurement of liver grafts (Figure 1B). Thus, induction of CD (CD group) did not modify protein expression of GLP1 levels in either intestine or plasma from donors with steatotic or non-steatotic livers when compared with the Sham group. However, in the CD group, hepatic GLP1 levels were lower in non-steatotic and higher in steatotic livers when compared with the Sham group. Plasmatic levels of DPP4 were similar in the Sham and the CD groups. In non-steatotic livers, hepatic levels of DPP4 were more elevated in the CD group than in the Sham group, whereas, in the presence of steatosis, DPP4 levels in the livers of the CD group were lower after CD induction than those observed in Sham group (Figure 1B).

### 3.3. Relevance of GLP1 in Non-Steatotic and Steatotic LT from DCDs

**Figure 2:**
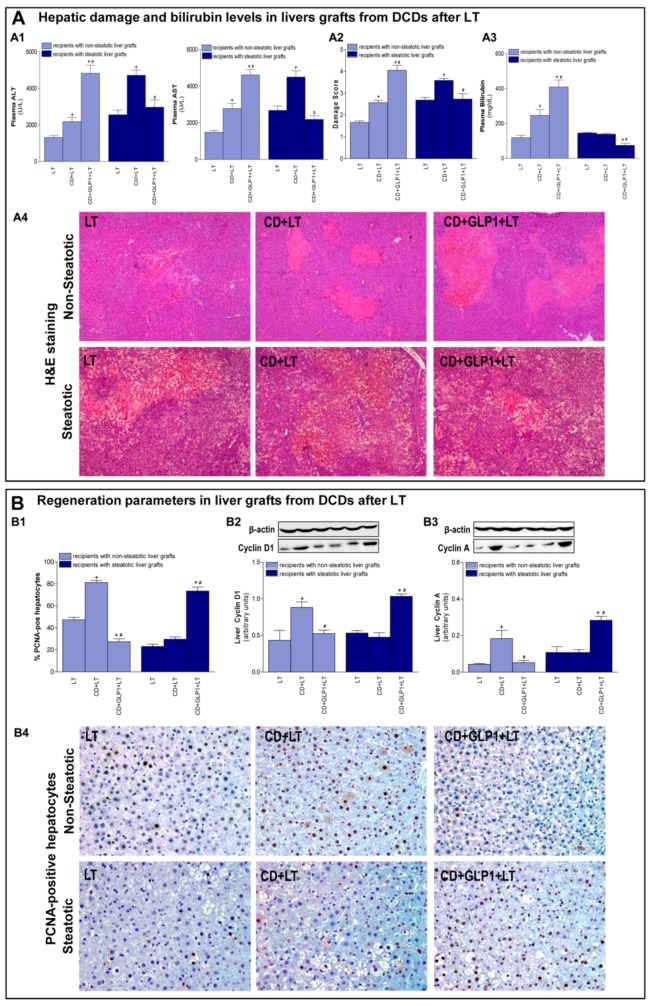
Liver injury and regeneration in non-steatotic and steatotic livers from DCDs after LT. (**A**) Hepatic damage parameters: plasma transaminases (**A1**), liver damage score (**A2**), bilirubin in plasma (**A3**), and representative photographs of histological changes in LT from DCDs (**A4**). In non-steatotic grafts, LT exhibited moderate and multifocal areas of coagulative necrosis, the extent and the number of necrotic areas being increased in CD+LT, whereas extensive and confluent areas of coagulative necrosis were observed in CD+GLP1+LT. In steatotic grafts, LT showed extensive and confluent areas of coagulative necrosis, whereas such coagulative necrosis was even more severe in CD+LT. The extent and the number of necrotic areas were reduced in CD+GLP1+LT when compared to CD+LT (4×). (**B**) Liver regeneration parameters: percentage of positive hepatocytes of anti-proliferating cell nuclear antigen (PCNA) (**B1**), protein expression of cyclin D1 (**B2**) and cyclin A (**B3**) in liver tissue, and representative photographs of immunohistochemical staining of PCNA-positive cells (**B4**). In non-steatotic livers, CD+LT showed an increased number of PCNA-positive hepatocytes compared to LT. CD+GLP1+LT showed fewer positive cells than CD+LT. In steatotic livers, CD+GLP1+LT showed an increased number of PCNA-positive hepatocytes compared to CD+LT (20×). For A1–A4 and B1–B4, six transplants per group were performed for each measurement. Representative Western blots for the proteins cyclins D1 and A and the housekeeping protein β-actin are shown at the top, and densitometric quantification of cyclins D1 and A protein expression normalized to β-actin (arbitrary units) is shown at the bottom. + *p* < 0.05 vs. LT; # *p* < 0.05 vs. CD+LT.

**Figure 3:**
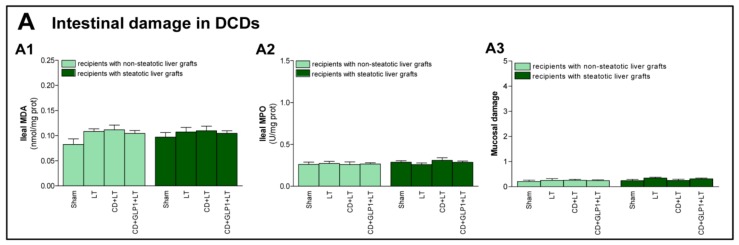
Intestinal damage in LT with DCDs. (**A**) Intestinal injury parameters: malondialdehyde (MDA) (**A1**), myeloperoxidase (MPO) (**A2**), and mucosal damage (**A3**) in intestine according to Chiu-score. For A1–A3, six transplants per group were performed for each measurement. Non-significant differences were observed in all groups evaluated.

First, we determined the effect of CD on damage and proliferation in non-steatotic and steatotic livers after transplantation. Transaminase levels and damage score values were higher in the CD+LT group than in the LT group (with non-CD donors) for both non-steatotic or steatotic liver grafts, with liver injury being greater in those with steatosis. In transplantation groups with non-steatotic grafts, total bilirubin in plasma was higher for CD+LT, while in the presence of steatosis, bilirubin levels were similar for both CD+LT and LT. Non-steatotic livers from the LT group showed moderate and multifocal areas of coagulative necrosis and neutrophil infiltration randomly distributed through the parenchyma, whereas the extent and the number of necrotic areas were higher in the CD+LT group. Steatotic grafts showed extensive and confluent areas of coagulative necrosis in the LT group, whereas such coagulative necrosis was even more severe for CD+LT (Figure 2A). With respect to liver regeneration, PCNA-positive cells and parameters of progression of the cellular cycle, such as levels of cyclin D1 (which is necessary for G1 phase progression) and cyclin A (which is necessary for S progression), were examined. In non-steatotic livers, there was an increment in PCNA-positive cells and in expression of cyclin D1 and A in the CD+LT group when compared to the LT group. In steatotic livers, such proliferation parameters were analogous in both CD+LT and LT groups (Figure 2B). CD did not appear to injure intestinal tissue considering that, in Sham, LT, and CD+LT experimental groups with either steatotic or non-steatotic liver, similar values of MDA, MPO, and histological Chiu score were observed (Figure 3).

To assess the relevance of GLP1 in non-steatotic and steatotic LT from DCDs, recombinant GLP1 infusion into the portal vein at a rate of 1 pMol × kg × min 10 min before CD was performed. The treatment with exogenous GLP1 resulted in increased levels of such protein in plasma and in liver from the CD+GLP1+LT group when compared to the CD+LT group for both non-steatotic and steatotic liver grafts (Figure 1A). In liver tissue, this increment was more evident in steatotic than in non-steatotic grafts. Alongside this, administration of GLP1 did not modify plasma DPP4 levels in the CD+LT group for any liver type. Regarding hepatic DPP4 levels, there was no difference between treated and untreated transplants from non-steatotic grafts from DCD (CD+GLP1+LT and CD+LT groups). However, DPP4 levels were reduced in steatotic grafts among the CD+GLP1+LT group compared to the CD+LT group (Figure 1A). In non-steatotic livers, treatment with recombinant GLP1 increased plasma transaminases, total bilirubin, damage score, and the extent of necrotic areas in the CD+GLP1+LT group compared to the CD+LT group (Figure 2A). In this type of liver, the number of PCNA-positive cells and the expression of cyclin D1 and A were lower in the CD+GLP1+LT group than in CD+LT (Figure 2B). In steatotic grafts, animals receiving GLP1 (CD+GLP1+LT) showed reduced transaminases and total bilirubin values compared to CD+LT. Such protection against damage in transplantation from DCDs was also evidenced by the histological study of steatotic livers, since there was a decrease in the damage score and in the extent and the number of necrotic areas in CD+GLP1+LT compared to CD+LT (Figure 2A). Administering GLP1 also caused a rise in the count of PCNA-positive cells and in levels of cyclin D1 and A when compared to grafts from CD donors without treatment in livers with steatosis (Figure 2B).

**Figure 4:**
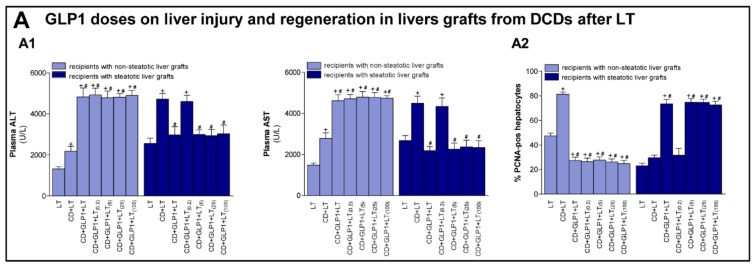
(**A**) Effect of GLP1 at different doses and administered 10 min before CD on liver injury and regeneration in non-steatotic and steatotic livers from DCDs after LT. The doses of GLP1 are the following: [GLP1 (1 pMol × kg × min), GLP1_(0.2)_ (0.2 pMol × kg × min), GLP1_(5)_ (5 pMol × kg × min), GLP1_(25)_ (25 pMol × kg × min) and GLP1_(100)_ (100 pMol × kg × min)]. Plasma transaminases (**A1**) and percentage of positive hepatocytes of PCNA (**A2**) in liver tissue. For A1–A2, six transplants per group were performed for each measurement. + *p* < 0.05 vs. LT; # *p* < 0.05 vs. CD+LT.

The dose and the pre-treatment time of GLP1 infusion at 1 pMol × kg × min 10 min before CD to evaluate the action mechanisms of GLP1 in both types of liver grafts was selected because lower and higher doses of GLP1 were not associated with improvements in the biochemical parameters of hepatic damage and regeneration (Figure 4). In the case of non-steatotic livers, all of the evaluated doses impaired hepatic injury and regeneration in transplanted rats. In addition, the pre-treatment time used in the present study (10 min before CD) resulted in parameters of hepatic injury and regeneration similar to those observed at longer pre-treatment times (30 or 60 min before surgical procedure) in both liver types (data not shown).

### 3.4. The Effect of GLP1 on Inflammatory Response in Non-Steatotic and Steatotic LT from DCDs

**Figure 5:**
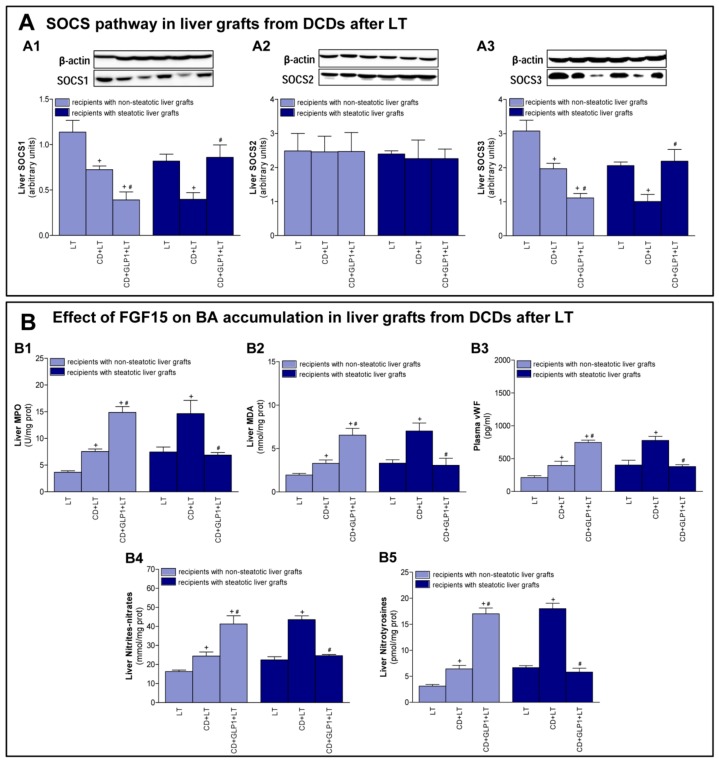
Effect of GLP1 on inflammatory responses in LT with DCDs. (**A**) Protein levels of suppressors of cytokine signaling 1 (SOCS1) (**A1**), SOCS2 (**A2**), and SOCS3 (**A3**) in liver. (**B**) MPO (**B1**) and MDA (**B2**) levels in liver, Von Willebrand Factor (vWF) in plasma (**B3**), and nitrate, nitrite (**B4**), and nitrotyrosine (**B5**) levels in liver. For A1–A3 and B1–B5, six transplants per group were performed for each measurement. Representative Western blots for the proteins SOCS1, SOCS2, and SOCS3 and the housekeeping protein β-actin are shown at the top, and densitometric quantification of SOCS1, SOCS2, and SOCS3 protein expression normalized to β-actin (arbitrary units) is shown at the bottom. + *p* < 0.05 vs. LT; # *p* < 0.05 vs. CD+LT.

Next, we investigated whether GLP1 effects are related to regulation of inflammatory responses. In non-steatotic grafts, SOCS1 and SOCS3 were lower in the CD+LT group than in the LT group, whereas SOCS2 was no different. Treatment with recombinant GLP1 (CD+GLP1+LT) decreased SOCS1 and SOCS3 expression in comparison to the CD+LT group (Figure 5A). This occurred in association with exacerbated inflammation parameters, as increased values of hepatic MPO, MDA, NO production (reflected in the values of tissue nitrates and nitrites), nitrotyrosine, and plasma vWF were registered in the CD+GLP1+LT group when compared to the CD+LT group (Figure 5B). Regarding steatotic grafts, CD induction appeared to affect SOCS1 and SOCS3 expression, since levels of these proteins were reduced in the CD+LT group when compared to the LT group, whereas SOCS2 was unmodified. When GLP1 was administered (CD+GLP1+LT), it resulted in higher SOCS1 and SOCS3 expression than in the CD+LT group, and this was accompanied by less liver inflammation, evidenced by decreased levels of liver MPO, MDA, NO production, nitrotyrosine, and plasma vWF (Figure 5A,B).

## 4. Discussion

Use of organs from DCDs is increasing in LT due to the persistent shortage of donors and waiting list mortality. However, several reports suggest inferior post-graft survival rates and an increased risk of primary non-function with such organs. Most studies agree that periods of warm ischemia prior to cold ischemia and hepatic steatosis contribute significantly to these aggressive hepatic complications [18,19,20]. In this study, we describe the beneficial effects of GLP1 treatment in LT from steatotic DCDs, as administration of GLP1 led to a reduction in hepatic damage and inflammation and improved the proliferative response.

Gut-derived GLP1 is rapidly degraded by DPP4 in the circulation, limiting its half-life [5]. Thus, GLP1 analogs, also known as GLP1 receptor (GLP1R) agonists (exendin-4 and liraglutide) have been used in order to regulate GLP1 actions in different liver diseases [21]. This is because GLP1R agonists (resistant to DPP4-mediated cleavage) are more stable and have a longer half-life in the circulation [21]. However, it should be considered that the use of recombinant GLP1, as we performed in the present study, offers several theoretical advantages over pharmacotherapy modalities based on GLP1 analogs. GLP1 exists in organisms in two forms, GLP1 (7–37) and GLP1 (7–36) amide, the latter being the most abundant in the circulation [22]. GLP1 analogs are molecules that have been modified to increase the half-life of GLP1, but this fact induces different effects to those of native GLP1. Indeed, it has been reported that the sustained GLP1R activation by these GLP1 analogs induces receptor desensitization [23]. Moreover, GLP1 analogs might exert functions different from those of native GLP1, including insulinotropic effects stimulating insulin secretion [21]. Thus, in order to resemble the in vivo conditions as much as possible, administering the recombinant native and active form of GLP1 (7–36) could be more suitable for using in investigations about effects of GLP1. Regarding disadvantageous physiological GLP1 degradation by DPP4, another way to counteract this that is different from the use of GLP1 analogous is through administering GLP1 recombinant intravenously via portal vein, as we made in the present research.

The dose and the timing of GLP1 infusion used in the present study were selected based on a previous study reported by Nishizawa M et al. [13]. There exist other studies that administered GLP1 in rats with the purpose of evaluating the metabolic effects of GLP1 regulation; generally in all these studies, a chronic [22,24,25] treatment with GLP1 was used. Only the study of Nishizawa et al. [13] administered GLP1 via portal vein for a short time (10 min) and was able to demonstrate an effect of such treatment on different metabolic pathways. It is well known that LT from a cadaveric donor is an emergency procedure in which there is very little time to pre-treat the donor with the different drugs. However, considering that the study of Nishizawa et al. did not evaluate liver injury, our research group performed preliminary dose-response studies. In our hands, we selected the dose of a drug that conferred with the highest protection against hepatic injury and regeneration at the shorter pre-treatment time (1 pMol × kg × min, portal vein, 10 min before CD). Regarding the use of intraportal infusion of GLP1 on clinical donor surgery, the translation of this therapeutic strategy from bench to bedside might be feasible. In line with this, it is common to perform intraportal infusions in liver surgery, including LT [26,27,28]. As shown in the present work, to counteract the deleterious effects of CD, GLP1 infusion is required to be administered in donors during a short time of 10 min before CD occurs. This could be clinically reasonable when CD occurs in controlled situations of cardiac arrest.

This is the first report showing the effects of GLP1 in LT with DCDs, which are quite different depending on the type of liver. In the present research, the administration of recombinant GLP1 in non-steatotic DCDs was associated with exacerbated damage, inflammation, and cell proliferation failure. Our results disagree with current research results that are mainly focused on administration of GLP1 analogs and which have demonstrated beneficial effects on non-steatotic livers suffering pathologies such as hepatic warm ischemia, thus suggesting a clinical importance for GLP1 as a therapeutic strategy [4]. The reason for the disparity in these results might be related to the type of ischemia involved, since it is well known that the molecular mechanisms implicated in warm hepatic I/R injury trigger different pathways than in cold I/R injury [29]. In addition, GLP1 analogues can act differently than native GLP1, as previously reported [21]. Herein, our results challenge the dogma about the protective properties of GLP1 as reported in the literature [21,30], since, in the case of non-steatotic LT from DCDs, treatment with GLP1 exerted harmful effects regarding damage and regeneration, indicating that it should be not considered an appropriate therapy in this context.

Discrepancies between hepatic GLP1 levels in steatotic and non-steatotic grafts when both are subjected to CD could be related to the DPP4 enzyme in livers. DPP4 is a serine protease that cleaves a variety of substrates, including GLP1 [31]. It is ubiquitously expressed on the apical surface of many cell types and occurs in soluble form (sDPP4) in the circulation [32,33]. There are numerous studies proving that sDPP4 degrades more than half of the circulating GLP1 [34,35,36]. In our view, the differences between GLP1 levels in steatotic and non-steatotic livers from DCDs might be explained by differences in hepatic DPP4 rather than by differences in either circulating GLP1 or DPP4. Indeed, circulating GLP1 and DPP4 levels were similar in both steatotic and non-steatotic DCDs. However, the low levels of GLP1 observed in non-steatotic livers were associated with an increase in the hepatic DPP4 enzyme that inactivates GLP1. Similarly, the high GLP1 levels observed in steatotic liver grafts were associated with a reduction in the hepatic enzyme, DPP4. Taken together, these results indicate that regulation of GLP1 levels in LT from DCDs is different depending on the type of liver. Thus, in our view, non-steatotic liver grafts trigger signaling that promotes an increase of DPP4, which in turn contributes to a decrease in local GLP1 availability in an attempt to limit the deleterious GLP1 effects on hepatic integrity and cell proliferation. On the other hand, the reduction in the expression of DPP4 in steatotic livers favors the accumulation of GLP1, which tries to counteract inflammatory responses, damage, and failure of cell proliferation observed in such types of liver when subjected to CD and LT. Given the above, it could be expected that, if GLP1 is administered in non-steatotic grafts from DCDs, DPP4 levels would be increased to inactivate such GLP1 and prevent its harmful effects. In the experimental cases evaluated here, there was no increase in DPP4 in non-steatotic grafts from DCDs to prevent the increase in GLP1 levels after administration of exogenous GLP1. Consequently, when GLP1 was administered in non-steatotic grafts, high hepatic GLP1 levels were observed, and this was associated with exacerbated damage, inflammatory responses, and proliferative failure, thus proving a deleterious role for GLP1 in non-steatotic grafts in LT from DCD. As we expected, regarding steatotic grafts from DCDs, following GLP1 administration, hepatic DPP4 was further reduced, and this triggered the accumulation of GLP1 in the liver, which is beneficially associated with diminished hepatic damage and inflammation and with enhanced cell proliferation. Thereby, we established that GLP1 plays a protective role in steatotic LT from DCDs. Steatotic livers have a more efficient GLP1 regulation system than non-steatotic livers, in which the DPP4 enzyme has a preponderant role. This mechanism of molecular control means that steatotic grafts reduce DPP4 levels, allowing the accumulation of exogenous GLP1, which can then exert its beneficial effects. In contrast, when non-steatotic grafts face an accumulation of exogenous GLP1, they are not able to increase the level of hepatic DPP4 and therefore cannot counteract the harmful effects of GLP1.

The mechanisms through which GLP1 produces differential effects on damage and regeneration in steatotic and non-steatotic grafts from DCDs were also evaluated. Endogenous SOCS proteins are key regulators of inflammatory response in different liver diseases and are essential for maintaining normal cell homeostasis [37]. The increased hepatic SOC1 and SOCS3 levels induced by GLP1 in steatotic LT from DCDs would decrease neutrophil accumulation, oxidative stress, and endothelial damage and consequently reduce damage and improve the proliferative response. Similarly, the reduced SOCS1 and SOCS3 levels induced by GLP1 in non-steatotic LT from DCDs might drive the exacerbated inflammation, damage, and regenerative failure. It is of great clinical relevance to design strategies aimed at reducing the injury induced by inflammatory responses, because it could increase the proliferative response. It is well known that inflammatory responses, including reactive oxygen species (ROS) and neutrophil accumulation, significantly induce DNA damage and reduce the capacity of liver regeneration [38,39,40,41]. In this context, a therapeutic strategy such as GLP1 administration, which regulates inflammation and reduces hepatic damage, could enhance cell proliferation in steatotic LT from DCDs. Conversely, treatment with GLP1 that heightens inflammatory response and worsens hepatic damage could impair cell proliferation in non-steatotic LT from DCDs.

**Figure 6:**
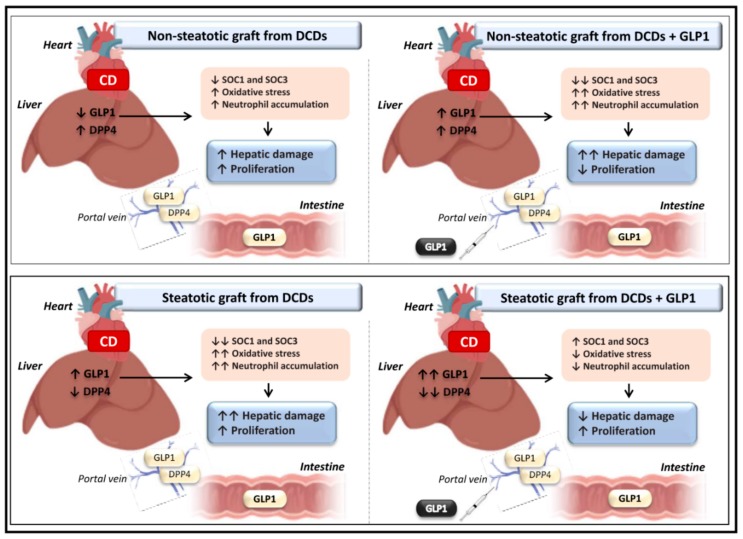
Schematic representation showing the effect of the different interventions (non-steatotic and steatotic liver grafts from DCDs with and without GLP1 administration) depicting outcomes and proposed signaling pathways of the current study.

In conclusion, the current study provides new mechanistic insights into the pathophysiology of LT from DCDs. Importantly, in LT from DCDs, different mechanisms that regulate endogenous GLP1 levels occur depending on the type of liver. We show the importance of preventing increases in GLP1 in non-steatotic liver grafts from DCDs to protect the grafts against damage, since GLP1 administration exacerbated damage in non-steatotic LT from DCDs (Figure 6). A different scenario occurred in the presence of hepatic steatosis, since this type of liver triggered the enzymatic machinery necessary for maintaining high levels of hepatic GLP1. Indeed, treatment with GLP1 protected steatotic liver grafts against inflammatory response, damage, and regenerative failure induced by CD. Notably, the results of these investigations are of clinical significance because grafts from DCDs are increasing in the clinical practice of LT and also because an increase in the percentage of donors with steatosis is expected due to the high prevalence of this disorder in society. Undoubtedly, additional intensive research will be necessary to evaluate whether the benefits of strategies based on GLP1 treatment, as observed in experimental models of LT using steatotic grafts from DCDs, can be transferred to clinical practice.
